# Radiofrequency ablation for inferior turbinate hypertrophy in patients with rhinitis medicamentosa

**DOI:** 10.1016/j.bjorl.2026.101895

**Published:** 2026-09-17

**Authors:** Narin N. Carmel-Neiderman, Ran Bilaus, Nidal Muhanna, Avraham Abergel

**Affiliations:** Tel Aviv University, School of Medicine, Tel Aviv Sourasky Medical Center, Department of Otolaryngology Head and Neck Surgery and Maxillofacial Surgery, Tel Aviv, Israel

**Keywords:** Rhinitis medicamentosa, Inferior turbinate hypertrophy, Radiofrequency ablation, Nasal obstruction, Quality of life

## Abstract

**Objectives:**

Nasal airway obstruction is commonly associated with Inferior Turbinate Hypertrophy (ITH), often stemming from chronic inflammation. Rhinitis Medicamentosa (RM), a type of non-allergic rhinitis, is frequently linked to prolonged use of nasal decongestants. We investigated Radiofrequency Ablation-assisted Turbinate Reduction (RFATR) as a second line in office treatment for RM, focusing upon its impact on decongestant spray cessation and Quality of Life (QoL).

**Methods:**

A prospective cohort study at a tertiary university-affiliated medical center. Consecutive adult patients with nasal airway obstruction and Inferior Turbinate Hypertrophy (ITH) who underwent Radiofrequency Ablation of the inferior Turbinates (RFATR). Patients were categorized into two groups: those with Rhinitis Medicamentosa (RM) and those without RM. The main outcome measures were cessation of nasal decongestant abuse, perioperative complications, and patient-reported responses to Quality-of-Life (QoL) questionnaires.

**Results:**

Fifty-three of the 256 patients who underwent RFA between March 2016 and March 2023 at our medical center were diagnosed as having RM. RM patients had a higher mean age and more comorbidities than non-RM patients. RM patients achieved significant improvements in He-NOSE questionnaires post-RFATR compared to non-RM patients (pre- and postoperative score difference of 11.9 ± 5.8 vs. 5.7 ± 6, respectively, p = 0.002). All RM patients ceased using decongestant spray use following RFATR. Complication rates and need for revision were low, with no significant group differences.

**Conclusions:**

RFATR is an effective and safe treatment for RM, significantly improving patients' QoL and facilitating decongestant spray cessation. Its minimal invasiveness, ease of administration, and compatibility with local anaesthesia make it a viable option for ceasing decongestant spray abuse with potential long-term benefits.

**Level of evidence:**

Level 3 evidence, according to the Oxford Centre for Evidence-Based Medicine 2011.

## Highlights

• RM is linked to prolonged nasal decongestant use.
• RFATR helps relieve nasal obstruction and ends spray abuse.
• 53/256 RFATR patients had RM; all stopped decongestant use.
• RM patients showed greater QoL improvement than non-RM.
• RFATR is a safe and effective in-office treatment for RM.

## Introduction

Nasal airway obstruction is a widespread medical condition often linked to Inferior Turbinate Hypertrophy (ITH).^1^^,^^2^ The underlying cause of ITH typically traces back to chronic inflammation within the nasal mucosa, culminating in mucosal hypertrophy and mechanical obstruction. This obstruction disrupts blood flow and compromises the regular functioning of the immune system, setting the stage for a self-perpetuating cycle of inflammation.^3^^,^^4^ ITH predominantly arises from prolonged rhinitis, which can be categorized into allergic (IgE-mediated) and non-allergic forms (attributed to factors such as infections, systemic ailments, atrophy or aging).^5^ Rhinitis Medicamentosa (RM) is a prevalent form of non-allergic rhinitis frequently linked to the protracted misuse of nasal decongestants. The pathophysiology of RM centers around the development of paradoxical nasal congestion following extended decongestant use, primarily via the activation of alpha-1A adrenoreceptors. While other medications, such as anti-psychotics, oral contraceptives, oral beta-adrenoceptor antagonists, anti-hypertensive drugs, and even cocaine can provoke rhinitis, topical decongestants are particularly notorious for their potent vasoconstrictive impact on the nasal mucosa.^6^^,^^7^

Histological alterations characteristic of RM include damage to the nasociliary apparatus, squamous cell metaplasia, epithelial edema, epithelial cell denudation, goblet cell hyperplasia, increased expression of the epidermal growth factor receptor and infiltration by inflammatory cells.^7^ The duration of exposure required to trigger RM was found to vary significantly, ranging from as early as 3-days up to 4‒6-weeks of continuous abuse of adrenergic spray.^8^ This illustrates the importance of counselling patients on the judicious use of such medications in order to minimize the risk of RM development.

Despite RM’s being a common entity in the rhinology outpatient clinic, the existing literature on its pathophysiology and therapeutic approaches and guidelines remains limited, with a dearth of prospective studies. Primary treatment modalities center upon discontinuing the abuse of decongestants, but, paradoxically, abrupt cessation may give rise to rebound congestion and patient frustration, potentially prompting a reinitiation of drug use.^9^

Radiofrequency Ablation-assisted Turbinate Reduction (RFATR) has emerged as an effective solution for ameliorating nasal breathing and enhancing the overall Quality of Life (QoL) for individuals contending with ITH. RFA involves the precise localized heating of submucosal tissue while preserving surface layers, leading to the induction of fibrosis during the subsequent healing process. Its appeal lies in its simplicity, non-invasiveness, and the ability to target specific areas, all while safeguarding the integrity of surrounding healthy tissue. An additional advantage is its compatibility with local anesthesia, enhancing its clinical utility among patients unable or unwilling to undergo surgery under general anesthesia.^1^, ^2^, ^3^, ^4^, ^5^, ^6^, ^7^, ^8^, ^9^, ^10^, ^11^, ^12^, ^13^, ^14^ Notably, RFA has shown effectiveness in a range of conditions, including ITH resulting from both allergic and non-allergic rhinitis,^13^^,^^15^, ^16^, ^17^, ^18^ and interestingly, concurrent mild-to-moderate septal deviation.^19^ Initial studies have shown unique results for RFA in patients with RM, such as enhancing their QoL and aiding in their cessation of adrenergic spray abuse.^20^

In this prospective study, therefore, we further examine RFA as a potential solution for cessation of adrenergic spray use and thereby enhancing the QoL of individuals presenting with RM. We describe our extended experience with patients with RM, examine their achievement in the cessation of decongestant medication use, and explore the potential for RFATR to serve as an alternative treatment solution for patients who failed conservative treatment.

## Methods

### Participants and design

This study is a prospective cohort investigation involving adults with nasal airway obstruction and ITH who underwent the RFATR procedure at the our institution between March 2016 and March 2023. Ethical approval for this study was obtained from the institutional Review Board (Approval Number: TLV-0367‐18). All RFATR procedures were conducted at the medical center by a single expert rhinologist.

Included were patients with a minimum follow-up of 2-months after RFATR who were 18-years of age and older, those diagnosed with ITH resulting from allergic and non-allergic rhinitis or RM, and those capable of providing informed consent and responding to questionnaires in Hebrew. Excluded were patients under 18-years of age, those lacking a minimum follow-up of 2-months after RFATR, pregnant women, patient’s incapable of providing informed consent to participate in the study for any reason, those whose responses to the questionnaires were inadequate, and those declining to enroll in the study. Also excluded were individuals with contraindications for RFATR treatment (e.g., contraindication to undergo local anaesthesia, presence of non-removable electronic implants susceptible to radiofrequency interference, such as pacemakers), those with a major sinonasal medical history (e.g., previous nasal malignancy, chronic sinusitis with or without polyposis, prior nasal surgeries), those with severe septal deviation causing limited visualization of the nasal cavity, and those previously exposed to radiation to the head and neck.

The data extracted from patient records encompassed medical history (e.g., ischemic heart disease, diabetes, hypertension, obstructive sleep apnea), a history of major nasal surgeries (e.g., septoplasty, conchotomy, rhinoplasty, rhinoseptoplasty), demographic characteristics, presenting symptoms, allergic complaints, smoking history, results of physical examinations, regular medication use, and imaging studies. An anonymous database was established for analysis. Patients were categorized into 2 groups based upon the underlying cause of rhinitis of RM or of both allergic and non-allergic rhinitis. In the absence of specific diagnostic criteria in the literature, we defined RM as the habitual use of a topical decongestant drug at least twice daily for a minimum of 2 consecutive months. Patients who failed to discontinue decongestant use were advised to gradually reduce the concentration of the decongestant and switch to a topical steroid spray. Patients whose symptoms did not resolve after adhering to this regimen were included in the study. All of the patients were followed up at 2- and 6-months post-procedure during outpatient clinic visits. Additionally, they all continued to receive telephonic follow-up queries, and those dissatisfied with their progress were reassessed at outpatient clinic visits.

### QOL assessment

Participants completed Quality of Life (QoL) questionnaires, including the validated Hebrew version of the Sino-Nasal Outcome Test-22 (He-SNOT-22^21^ and the Nasal Obstruction Symptom Evaluation (He-NOSE),^22^ both before the Radiofrequency Ablation (RFA) procedure (baseline) and after the procedure. Postoperative SNOT-22 scores along with the patients' overall subjective impressions were assessed at the 2-month post-RFATR follow-up.

#### He-SNOT-22 questionnaire

An overall QoL value assessed by the Minimal Clinically Important Difference (MCID) as was established at 8.9 for QoL measures.^23^ We calculated the proportion of patients in each group who achieved an MCID of at least an 8.9-point improvement on the SNOT-22 questionnaire. A validated translation of SNOT-22 to Hebrew^21^ version was used.

#### He-NOSE questionnaire

Originally developed and validated in 2004, the Nasal Obstruction Symptom Evaluation (NOSE) questionnaire serves as a valuable tool for evaluating the impact of nasal obstruction on the QoL among adults. We ensured the validity of the Hebrew version of the questionnaire through a meticulous process of translation and re-translation. An independent cross-cultural adaptation and validation of the He-NOSE scale has further confirmed its psychometric properties in Hebrew-speaking patients.^24^ Following validation within our rhinology clinic's patient population, we added the NOSE questionnaire to the SNOT-22 questionnaire. This addition allowed us to specifically assess nasal obstruction-related symptoms and their influence on overall QoL.

### Surgical technique

**Fig. 2:**
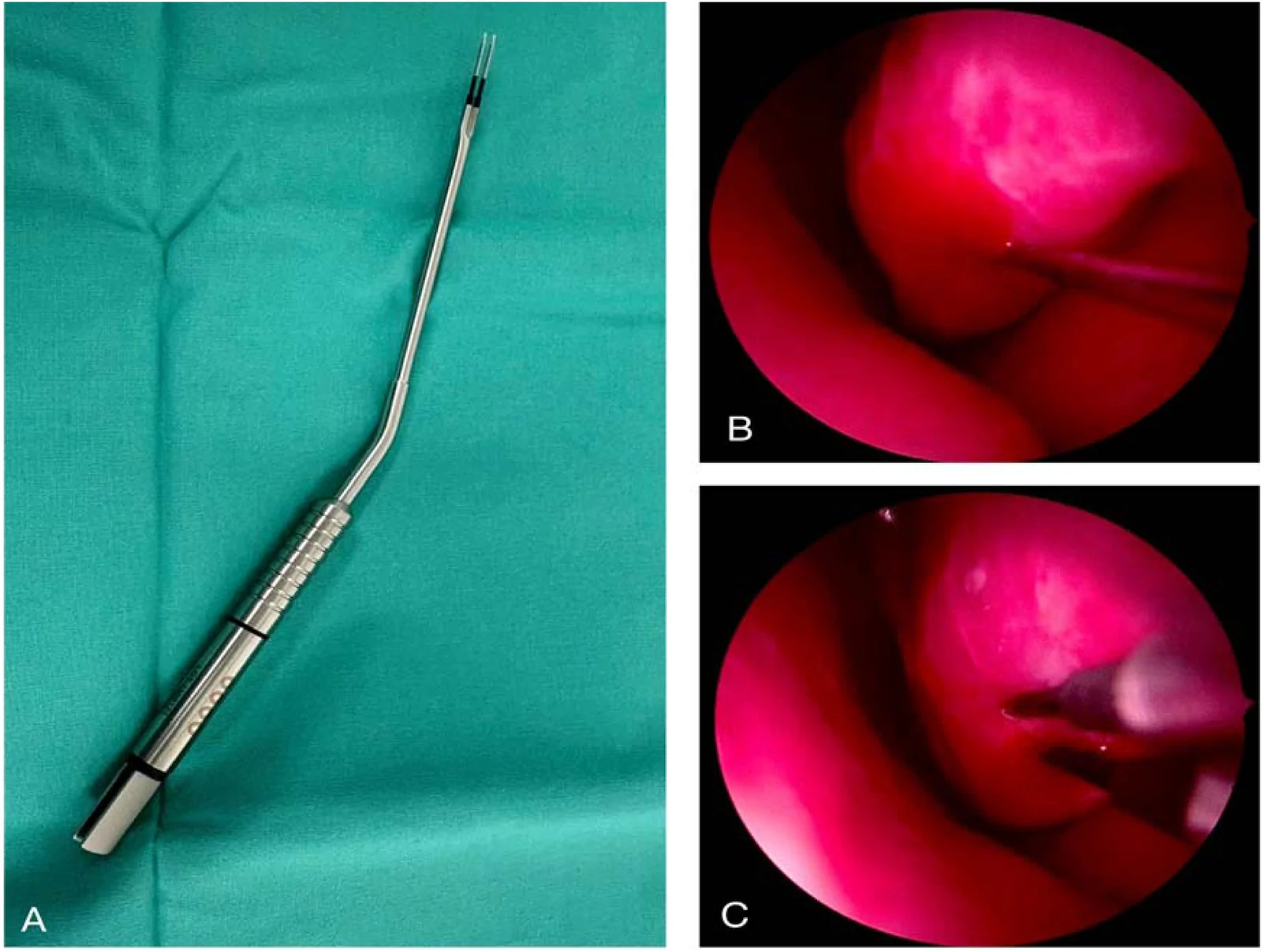
(A) Bipolar radiofrequency needle electrode (Aftmost, Inomed Ltd, Germany). (B) Injection of lidocaine into the inferior turbinate under endoscopic guidance. (C) Submucosal delivery of radiofrequency energy during the RFATR procedure.

Local anesthesia was administered by placing a cotton pledget saturated with a 2% tetracaine solution mixed with oxymetazoline (in a 1:1 ratio) in the middle and inferior meatus for a duration of 15–20 min. Subsequently, under endoscopic guidance, 5 cc of 2% lidocaine was injected into the inferior turbinates. Turbinate reduction was achieved by using a bipolar radiofrequency electrode. The device is a bipolar radiofrequency generator (Aftmost, Inomed Ltd, Germany, 15 W automated mode) that delivers controlled radiofrequency energy at a frequency of 500 kHz through a bipolar needle electrode. Energy was applied submucosally at each of four to five predefined points along the inferior turbinate bilaterally, with an average application time of approximately 10 seconds per lesion, resulting in a tissue temperature of approximately 40–70 °C, creating 4–5 lesions along the inferior turbinate on both sides (Fig. 2. Lateralization of the turbinates was performed when necessary. Revision RFA followed a similar procedure.

Following the intervention, patients were instructed to perform high-flow nasal rinses with 0.9% saline solution per nostril. These rinses commenced 1–2 days after the surgery, and either a bottle or a syringe was used to administer the solution into the nose 3–4 times daily for a period of 2-weeks. The frequency of application was then reduced to 1–2 times daily until the first postoperative clinic visit, which took place 2-months after the surgery.

### Statistical analysis

Categorical variables were summarized as frequency and percentage. Continuous variable distribution was evaluated using a histogram, and all continuous variables were reported as median and interquartile range since they were not normally distributed. Categorical variables were compared between groups using the Chi-Squared test or Fisher's exact test, and continuous variables were compared using the Mann-Whitney test. A Kaplan Meir curve and the Log Rank test were used to describe and compare any events during the follow-up period. A reverse centering method was used to evaluate the length of follow-up. All of the statistical tests were 2‐sided, and statistical significance was defined by a p-value < 0.05. All statistical analyses were performed with SPSS software (IBM SPSS statistics for Windows, version 28; IBM Corp, New York, USA, 2021).

## Results

The study comprised a cohort of 256 patients who underwent RFA between March 2016 and March 2023 in the Tel-Aviv Sourasky Medical Center, of whom 53 (20.7%) were diagnosed as having RM.

### Study cohort

Patients with RM had a notably higher average age (49.9 ± 18.3 years) compared to the non-RM group (39.4 ± 18.3 years, p < 0.001). There was a male predominance of 66% (n = 170) in both groups with no significant group differences (p = 0.95). Nearly one-half of the RM group reported a medical background compared to only one-quarter of the non-RM group (n = 51 [25.1%] vs. n = 26 [49.1%], respectively, p > 0.001). Hypertension was more prevalent in the RM group compared to the non-RM group (n = 11 [20.8%] vs. n = 16 [7.9%], p = 0.007). Other medical comorbidities, such as diabetes mellitus, obstructive sleep apnea, and autoimmune history, were comparable between the groups (Table 1). The RM patients reported fewer symptoms of nasal secretions compared to the non-RM patients (n = 9 [17%] vs. n = 62 [30.5%], p = 0.05). Edematous mucosa was more prevalent in the RM group compared to the non-RM group (n = 18 [34%] vs. n = 37 [18.2%], p = 0.013). Differences in the findings on physical examination of septal deviation, middle and inferior turbinate hypertrophy, presence of polypoid mucosa and sinus discharge were non-significant among the 2 groups.

**Table 1:** Demographics and characteristics of patients with RM *vs.* patients without RM.

	Rhinitis Medicamentosa (n = 53)	Non-Rhinitis Medicamentosa (n = 203)		Cohort (n = 256)
Parameter	n (%)	n (%)	p-value	n (%)
**Background**				
Age (average ± SD)	49.9 ± 18.3	39.4 ± 18.3	**<0.001**	41.6 ± 18.8
(median [IQR])	47 (34.5–69)	35 (23–52)		36.5 (25.75–57)
Male	35 (66)	135 (66.5)	0.95	170 (66.4)
Female	18 (34)	68 (33.5)	0.95	86 (33.6)
Smoking	7 (13.2)	12 (5.9)	0.08	19 (7.4)
Medical history	**26 (49.1)**	51 (25.1)	**<0.001**	77 (30.1)
Hypertension	**11 (20.8)**	16 (7.9)	**0.007**	27 (10.5)
Diabetes mellitus	3 (5.7%)	6 (3)	0.39	9 (3.5)
Obstructive sleep apnea	1 (1.9)	19 (9.4)	0.09	20 (7.8)
Autoimmune history	2 (3.8)	4 (2)	0.61	6 (2.3)
Allergic symptoms	8 (15.1)	52 (25.6)	0.11	60 (23.4)
Diagnosed allergy	2 (3.8)	**36 (17.7)**	**0.011**	38 (14.8)
Asthma	3 (5.7)	6 (3)	0.39	9 (3.5)
Nasal fracture or contusion	3 (5.7)	7 (3.4)	0.44	10 (3.9)
**Symptoms**				
Nasal Secretions	9 (17)	**62 (30.5)**	**0.05**	71 (27.7)
Snoring	11 (20.8)	43 (21.2)	0.95	54 (21.1)
Anosmia or parosmia	2 (3.8)	20 (9.9)	0.27	22 (8.6)
**Prior medical or surgical interventions**				
Nasal operation	8 (15.1)	45 (22.2)	0.26	53 (20.7)
Septoplasty ± conchotomy	7 (13.2)	36 (17.7)	0.43	43 (16.8)
Rhinoplasty	1 (1.9)	14 (6.9)	0.32	15 (5.9)
FESS	0 (0)	6 (3)	0.35	6 (2.3)
Topical nasal spray	**53 (100)**	60 (29.6)	**<0.001**	113 (44.1)
Oral antihistamines	0 (0)	**18 (8.9)**	**0.029**	18 (7)
Oral steroids	1 (1.9)	2 (1)	0.50	3 (1.2)
**Physical examination**				
Deviated nasal septum	36 (67.9)	147 (72.4)	0.52	183 (71.5)
Middle turbinate hypertrophy	6 (11.3)	16 (7.9)	0.42	22 (8.6)
Inferior turbinate hypertrophy	49 (92.5)	191 (94.1)	0.75	240 (93.8)
Edematous mucosa	**18 (34)**	37 (18.2)	**0.013**	55 (21.5)
Polypoid mucosa	2 (3.8)	6 (3)	0.67	8 (3.1)
Discharge from sinus	1 (1.9)	5 (2.5)	>0.99	6 (2.3)
**Prior radiological studies**				
Computed tomography	6 (11.3)	29 (14.3)	0.58	35 (13.7)
Magnetic resonance imaging	0 (0)	3 (1.5)	>0.99	3 (1.2)
**Postoperative**				
Subjective improvement	35 (79.5)	135 (81.3)	0.79	170 (81)
Complications	3 (5.7)	2 (1)	0.06	5 (2)
Revision (within 5-years)	9 (19.1)	21 (14.7)	0.23	30 (11.7)
Achieved MCID (SNOT22)	16 (66.7)	41 (52.6)	0.22	57 (55.9)

FESS, Functional Endoscopic Sinus Surgery; MCID, Minimal Clinically Important Differences; SD, Standard Deviation; SNOT, Sinonasal Outcome Test; IQR, Interquartile Range.

**Fig. 1:**
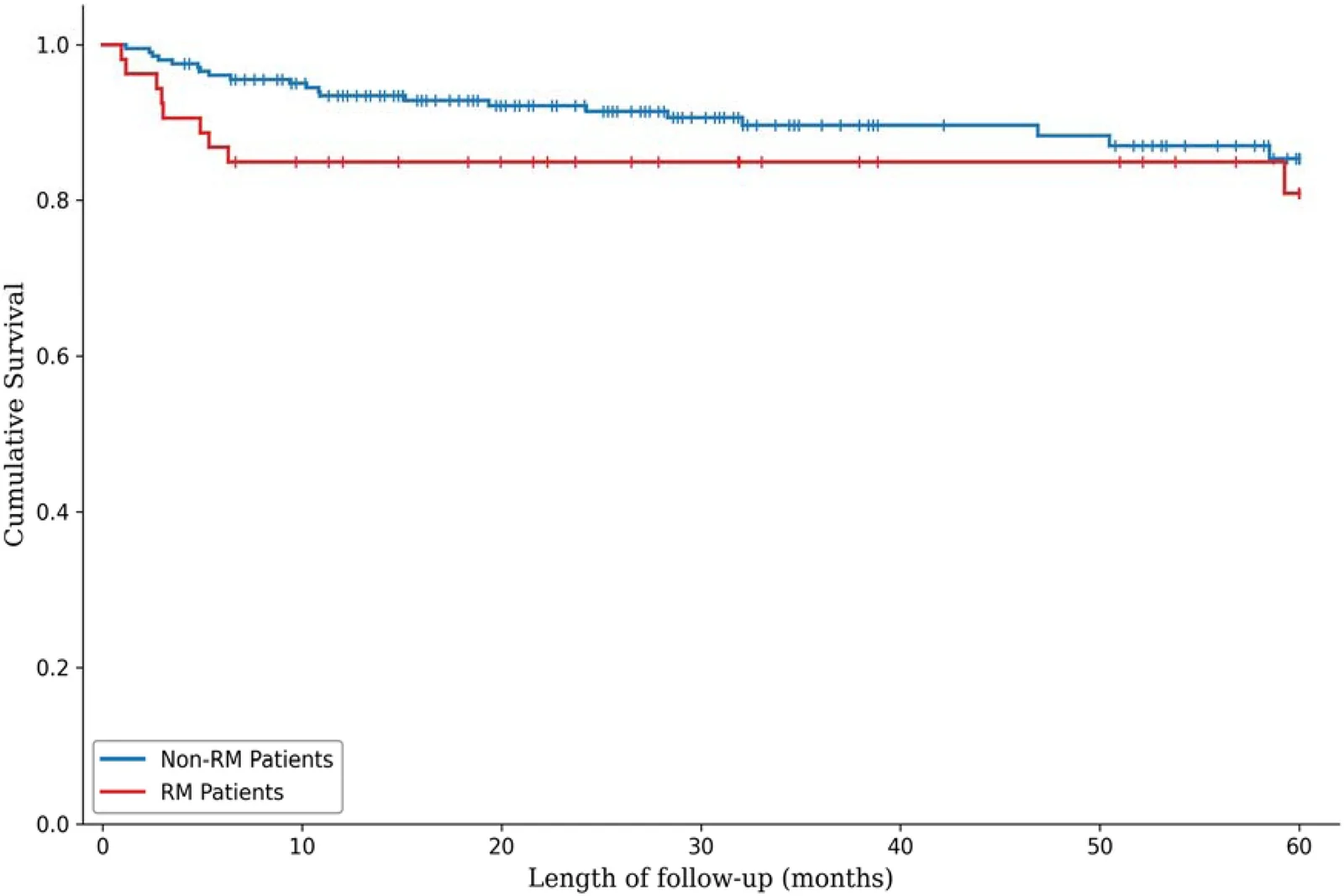
Kaplan-Meier curve of revision events throughout 60-months of follow-up.

Both groups had high levels of reported patient satisfaction following the procedure, with low rates of postoperative complications or need for revision (p = NS for the 2 groups) (Fig. 1). Notably, all 53 RM patients ceased decongestant spray use following the procedure.

### Quality of Life

#### He-NOSE questionnaire

Thirteen RM patients and 65 non-RM group completed the He-NOSE questionnaire. The RM group's mean preoperative score of 16.1 ± 4.1 was significantly higher compared to the non-RM group’s mean score of 13 ± 4.1 (p = 0.007). Following the procedure, the RM group's mean score was 4.1 ± 3.8 compared to the non-RM group’s a mean score of 7.3 ± 5.2 (p = 0.036). Examination of the postoperative score differences revealed that the RM group experienced a significantly greater improvement (mean 11.9 ± 5.8) compared to the non-RM group (mean 5.7 ± 6, p = 0.002).

#### He-SNOT-22 questionnaire

Data were retrieved from 24 RM patients and 78 non-RM patients. The former group had a mean preoperative score of 32.2 ± 17.4 and a mean postoperative score of 14.3 ± 14.7, while the latter group had a mean preoperative score of 38.5 ± 20.2 and a mean postoperative score of 27.1 ± 21.5. The group differences in the postoperative scores were highly significant (p *=* 0.002). Assessment of the differences in scores before and after the operation, however, revealed no significant group differences.

## Discussion

We had earlier reported the initial findings on efficacy and safety of RFA performed on 24 patients between March 2016 and June 2019, with resultant high rates of decongestant spray cessation, thus portending a promising long-term outcome.^19^ Our current study extends our experience with RFA in RM patients to March 2023, and the results reveal gratifying enduring effects. The current cohort was expanded to include 53 RM patients who had low rates of revision and complications post-RFA, coupled with high patient satisfaction, thus demonstrating the procedure's long-term safety and efficacy in the cessation of decongestant abuse. Notably, none of the 53 RM patients resumed decongestant spray use throughout the study period, allowing for treatment of the underlying cause of the RM.

While there is currently no standardized treatment protocol for the treatment of RM, current guidelines recommend withdrawal of decongestant spray use whose efficacy remains questionable and fails to address the underlying cause of the nasal congestion.^6^^,^^25^^,^^26^ The RFA protocol provides both a treatment for RM that targets the nasal mucosa and addresses the underlying condition causing the nasal congestion, in addition to initiating and maintaining the withdrawal process.

Interestingly, 8 of the 9 patients who required any revision following the procedure underwent revision RFA alone, and none required further intervention. None of them sustained any complications, and none resumed the use of decongestant spray. This finding suggests that RFA could be used to further treat any residual mucosal disease among RM patients with persistent nasal obstruction following initial RFA by means of a minimally invasive in-office procedure.

A novel finding in this study is the impressive improvement in the QoL measures among RM patients post-RFA, further demonstrating significant benefit derived from this intervention. The He-NOSE questionnaire scores revealed significant higher preoperative scores (16.1 ± 4.1 in the RM group vs. 13 ± 4.1 in the non-RM group, p = 0.007) indicative of major QoL impairment due to RM. Correspondingly, the postoperative scores were significantly lower for the RM group (4.1 ± 3.8 vs. 7.3 ± 5.2 in the non-RM group, p *=* 0.036), indicating a substantial improvement in QoL following the procedure in the RM patients with RM compared to the non-RM patients. Furthermore, the RM group exhibited a significantly higher difference between the pre- and postoperative scores (11.9 ± 5.8 vs. 5.7 ± 6 for the non-RM group, p = 0.002), indicating a marked improvement in QoL following RFA in the former group and high level of efficacy in alleviating symptoms of nasal obstruction due to RM (Table 2).

**Table 2:** Scores on QoL questionnaires in patients with RM *vs* patients without RM.

	Rhinitis Medicamentosa (average ± SD)	Non-rhinitis Medicamentosa (average ± SD)	
Questionnaire scores	(median [IQR])	(median [IQR])	p-value
**He-SNOT-22**			
	n = 24	n = 78	
Preoperative score	32.2 ± 17.4	38.5 ± 20.2	0.21
	34 (15.75–47.25)	37.5 (23.75–51.87)	
Postoperative score	14.3 ± 14.7	27.1 ± 21.5	**0.002**
	8.5 (4.25–21.75)	21 (11–39)	
Score difference	17.9 ± 17.7	11.4 ± 16.7	0.16
	15 (4.5–30.75)	11 (0.75–22.25)	
**He-NOSE**			
	n = 13	n = 65	
Preoperative score	16.1 ± 4.1	13 ± 4.1	**0.007**
	17 (15–19)	13 (10–16)	
Postoperative score	4.1 ± 3.8	7.3 ± 5.2	**0.036**
	3 (0.5–8)	6 (3–10.5)	
Score difference	11.9 ± 5.8	5.7 ± 6	**0.002**
	12 (7.5–17)	5 (1.5–10)	

SNOT, Sinonasal Outcome Test; NOSE, Nasal Obstruction and Septoplasty Effectiveness; IQR, Interquartile Range; SD, Standard Deviation.

There had been no significant group difference in the preoperative He-SNOT questionnaire scores, but the postoperative scores were significantly lower in the RM group (14.3 ± 14.7 vs. 27.1 ± 21.5 in the non-RM group, p = 0.002). However, the difference between the pre- and postoperative scores was non-significant. Moreover, the proportion of patients who reached the MCID was comparable between the 2 groups. It should be borne in mind that the SNOT-22 questionnaire had initially been intended for rhinosinusitis and that it reflects shared systemic characteristics due to the common T-helper type-2 mechanism underlying both conditions.^27^^,^^28^ Conversely, the NOSE questionnaire was tailored specifically for nasal obstruction-related symptoms. This distinction in the design and focus of these tools may have impacted the observed results, potentially accounting for the varying outcomes in scores.

There were noteworthy demographic and clinical differences between the RM and non-RM patients. The RM patients were older on average compared to the non-RM group (49.9±18.3 vs. 39.4±18.3 years of age, p < 0.001). In addition, a significant proportion of RM patients (49.1%, n = 26) had comorbidities compared to the non-RM group (25.1%, n = 51) (p < 0.001). These findings may support the preference for RFA, a minimally invasive procedure performed in-office under local anesthesia, for patients who are less favorable surgical candidates.

Diagnosed allergies were less common among the RM patients (3.8%, n = 2) compared to the non-RM group (17.7%, n = 36) (p = 0.011). Perhaps, RM patients suffer from non-allergic rhinitis, attributed to their medical background, including regular medical regime, that is not possible to cease. In contrast, and as expected, edematous mucosa was observed more frequently among the RM patients (34%, n = 18) compared to the non-RM patients (18.2%, n = 37, p = 0.013), reflecting the pathophysiological changes typically seen after prolonged adrenergic spray abuse in patients with RM.^29^^,^^30^ Perhaps allergic patients had better conservative solutions for rhinitis, and therefore did not abuse decongestants.

Our study has certain limitations. Primarily, it does not conclusively determine whether or not RFATR directly affects RM pathophysiology or if the improved withdrawal rates are due to lifestyle changes post-surgery. Therefore, the definitive reason for the superior effectiveness and higher withdrawal rates compared to other treatments has yet to be established. Additionally, the use of questionnaires for QoL assessment could introduce reporting bias. The Hebrew version of the Nasal Obstruction Symptom Evaluation (He-NOSE) questionnaire had not yet been validated in the initial stages of the study. Consequently, we utilized the Hebrew Sino-Nasal Outcome Test-22 (He-SNOT-22) questionnaire, which is primarily tailored for chronic rhinosinusitis rather than specifically for rhinitis and nasal obstruction. Moreover, the choice of both the initial treatment and the revision in this prospective cohort study was made together with the patients who were not randomized to different treatments. Finally, the study’s having been conducted in a single tertiary center may limit the generalizability of the findings. Furthermore, the comparison between RM and non-RM patients should be interpreted with caution, as the non-RM group comprised a heterogeneous population including patients with allergic rhinitis and other etiologies of ITH. Since RFATR addresses the volumetric component of turbinate hypertrophy rather than the underlying inflammatory or allergic pathophysiology, the treatment effect may differ across these subgroups. This heterogeneity may introduce confounding bias when comparing outcomes between the two groups. Future studies with more homogeneous control groups may help to further delineate the specific efficacy of RFATR in RM patients.

## Conclusions

The findings of this study demonstrate the long-term effectiveness and safety of RFATR in treating RM. The procedure led to the cessation of decongestant spray use among all of the RM patients, and significantly improved their QoL. RFATR should be considered as a safe and effective second-line treatment option for patients with rhinitis medicamentosa who have failed conservative treatment, offering both symptom relief and facilitation of decongestant withdrawal.

## ORCID ID

Narin N. Carmel-Neiderman: 0000-0003-0628-1942

Nidal Muhanna: 0000-0003-1120-1062

Avraham Abergel: 0000-0002-9360-8888

## Ethical approval

The study was approved by the local ethical committee (0367‐18‐TLV).

## Data availability statement

The authors declare that all data are available in repository.

## Declaration of competing interest

The authors declare no conflicts of interest.
